# Association between multidrug-resistant bacteria and outcomes in intensive care unit patients: a non-interventional study

**DOI:** 10.3389/fpubh.2023.1297350

**Published:** 2024-01-08

**Authors:** Alessandro Pacheco Silveira Martins, Camila Pacheco Silveira Martins da Mata, Uener Ribeiro dos Santos, César Augusto de Araújo, Edna Marilea Meireiles Leite, Luciana Debortoli de Carvalho, Pedro Guatimosim Vidigal, Cristina Dutra Vieira, Simone Gonçalves dos Santos-Key

**Affiliations:** ^1^Hospital Risoleta Tolentino Neves, Universidade Federal de Minas Gerais, Belo Horizonte, Brazil; ^2^Departamento de Microbiologia, Instituto de Ciências Biológicas, Universidade Federal de Minas Gerais, Belo Horizonte, Brazil; ^3^Departmento de Ciências Biológicas, Universidade Estadual de Santa Cruz, Ilhéus, Brazil; ^4^Escola de Medicina, Departmento de Patologia Clínica, Universidade Federal de Minas Gerais, Belo Horizonte, Brazil

**Keywords:** healthcare-associated infection, intensive care unit, multidrug-resistant bacteria, active surveillance cultures, invasive devices

## Abstract

**Background:**

In intensive care units (ICUs), infections by multidrug-resistant (MDR) microorganisms should be monitored to prevent healthcare-associated infections (HAIs).

**Methods:**

From 2018 to 2020, we investigated all medical records of patients admitted to the ICU of a public university hospital. All patients colonized/infected by MDR microorganisms and submitted to active surveillance cultures (ASCs) were included.

**Results and discussion:**

Male patients prevailed, and 9.5% were positive for MDR bacteria. In-hospital deaths were statistically significant (*p* < 0.05) for older patients, patients with orotracheal tube use during previous and current hospitalization, and patients with high blood pressure, cardiac and pulmonary diseases, and chronic kidney disease. Carbapenem resistant Enterobacteriaceae was the most frequently resistance profile, followed by extended-spectrum beta-lactamase. The diagnosis or evolution of HAIs was statistically significant (*p* < 0.0001) for patients treated with meropenem and vancomycin, and in-hospital deaths occurred in 29.5% of patients using polypeptides while the use of macrolides reduced the odds for mortality. The BRADEN Scale demonstrated that 50% of the patients were at high risk of dying.

**Conclusion:**

Patients hospitalized in the ICU, colonized or infected by MDR bacteria, using invasive medical devices, and with underlying medical conditions presented increased mortality rates. The prescription of meropenem and vancomycin should be carefully monitored once patients using these antimicrobials already have or develop an HAI.

## 1 Introduction

Healthcare-associated infections (HAIs) are related to medical assistance, and patients in intensive care units (ICUs) present higher mortality rates, especially when associated with HAIs and invasive medical devices. In the ICU, patients are critically ill, receive more complex antimicrobial treatments, and are exposed to multidrug-resistant (MDR) microorganisms (1).

During the last decade, the family *Enterobacteriaceae* was considered the main cause of nosocomial infections due to their ubiquity and resistance profile (2). Some members of this family are included in the ESKAPE group (*Enterococcus*, *S. aureus*, *K. pneumoniae*, *A. baumannii*, *P. aeruginosa*, and *E. coli*) and can cause economic impact (3, 4), and increase healthcare institutions’ burden (5, 6). Monitoring the microbiota of ICU patients using active surveillance cultures (ASCs) could prevent the occurrence of HAIs by early identification of colonized or infected patients. Based on ASC results, it is also possible to establish routines and guidelines to control and reduce the risk of HAI (7). The performance of ASCs should also benefit patients and medical institutions, preventing in-hospital deaths. This study aimed to evaluate and statistically compare the outcome of patients in the ICU, submitted to ASC, and positive for MDR microorganisms.

## 2 Materials and methods

### 2.1 Population and hospital characteristics

Our sample was composed of patients admitted to the ICU of a university public hospital from January 1, 2018, to December 31, 2020. The ICU receives monthly 120 adult patients *circa* from emergency room, surgical and clinical wards. In the present study, patients from General Clinic prevailed (40.0%) and were followed by vascular surgery (24.0%). The detailed epidemiological data of the patients, the leading recovered microorganisms, and the hospital aspects were previously published (8). All medical records identifying the presence of MDR bacteria and its resistance profile during ASC procedures were registered.

### 2.2 Other investigated aspects

#### 2.2.1 Severity of illness scores during admission

During hospital admission, clinical conditions were recorded by a multidisciplinary team. These parameters established the severity of illness and the possibility of in-hospital death (9). Four indexes were adopted: Acute Physiology and Chronic Health Evaluation II (APACHE II), which can predict the diseases’ severity and the possibility of in-hospital mortality (10); Sequential Organ Failure Assessment (SOFA) that determines the extension of an organ function or rate of failure (9); Therapeutic Intervention Scoring System-28 (TISS-28) that measures the severity of patients admission, the nursing workload, and all therapeutic interventions and procedures (11, 12); and the BRADEN Scale that measures the risk for pressure ulcers (11).

#### 2.2.2 Presence of invasive medical devices and antimicrobial use

The use of an indwelling urinary catheter (IUC), central venous catheter (CVC), mechanical ventilation, and/or endotracheal ventilation were noted. Nine groups of antimicrobials were registered: ß-lactams, aminoglycosides, glycopeptides, polypeptides, macrolides, quinolones, sulfonamides, lincosamides, and nitroimidazoles.

#### 2.2.3 Outcome

Two possible outcomes were reported: death or hospital discharge. It is important to highlight that the same patient was admitted more than once.

#### 2.2.4 Antimicrobial resistance profile

All bacteria isolated from ASC were identified by conventional methods and by automated system Vitek 2 (BioMérieux, Marcy l’Etoile, France). Antimicrobial resistance profile was obtained by disk-diffusion (Kirby-Bauer) of antimicrobial agents selected by the hospital committee and include these nine classes: β-lactam, glycopeptides, polypeptides, nitroimidazoles, aminoglycosides, macrolides, quinolones, and lincosamides. In this study, multidrug-resistant microorganisms were those resistant to more than one antimicrobial from three different classes (13). The resistance profile of all strains was recorded and included ESBL-producing *Enterobacteriaceae*, *Klebsiella pneumoniae* carbapenemases producing (KPC), *Klebsiella pneumoniae* carbapenemase producing/polymyxin resistant strains (KPC/POLY), detection of multidrug resistant microorganism (MR), *Staphylococcus aureus* methicillin-resistant (MRSA), carbapenem resistant *Enterobacteriaceae* (CRE), and vancomycin-resistant Enterococci (VRE).

### 2.3 Statistical analysis

All statistical analyses were performed using GraphPad Prism software version 9.0.0 (San Diego, CA, United States). Data included age and gender of all patients, previous hospitalization (same or another hospital), isolation of MDR bacteria (colonization/infection), severity illness scores, previous ICU stay (same or another hospital), use of invasive devices, presence of HAI due to medical procedures, and antimicrobial use. The formula Sensitivity (S) determined the true positive results of ASCs with positive MDR bacteria.


$$S=S^{+}C^{+}∕S^{-}C^{+}$$

*S^+^C^+^*: positive swab results related to a positive culture; S^−^C^+^: negative swab results with a subsequent positive culture.

The positive predictive value (PPV) was *calculated* according to the formula:


$$PPV=S^{+}C^{+}/\left(S^{+}C^{+}\right)+\left(S^{+}C^{-}\right)$$

*S^+^C^+^*: positive swab results related to a positive culture; *S^+^C^−^*: the positive swab result was followed by a negative culture.

Bivariate analysis using the chi-square test of Pearson (*X*^2^) and Fisher’s exact test (significance level of 5%, *p* < 0.05) were used to investigate the association between age, gender, ICU admission, and previous hospitalization, with a positive ASC and the microorganisms recovered from clinical specimens considering the outcomes. Data were represented by absolute frequencies (*n*), percentage (%), odds ratio (OR), 95% confidence interval (95% CI), and the respective *p* values.

The Shapiro–Wilk test evaluated the normality of the quantitative variables. One-way ANOVA test followed by Tukey’s post-test for multiple comparisons investigated those variables with a normal distribution. Variables that did not assume a normal distribution were analyzed by the Mann–Whitney and the Kruskal–Wallis tests, followed by Dunn’s post-test for multiple comparisons, with a significance level of 5% (*p* < 0.05). Data were presented as individual values, mean, ± standard deviation (SD), minimum, maximum, and median values associated with the respective *p* values.

### 2.4 Ethical aspects

This research was approved by the Educational and Extension Center of the Risoleta Tolentino Neves Hospital (NEPE/HRTN #22/2018) and the Ethics and Research Committee of the Federal University of Minas Gerais (CEP/UFMG—CAAE: 39871820.1.0000.5149).

## 3 Results

### 3.1 Evaluation of ASC performance

The highest PPV *for MDR bacteria was Pseudomonas* spp. (15.4%), followed by *Acinetobacter* spp. (12.6%), and *Proteus* spp. (12.5%). The results demonstrated that the performance of ASC was an ineffective predictor for HAIs. *Acinetobacter* spp. presented the highest values of sensitivity (19.6%), followed by *Escherichia* spp. (10.0%), and *Pseudomonas* spp. (9.5%). These results also indicated that ASCs were not able to predict the risk of a future HAI.

### 3.2 Characteristics of hospitalized patients and hospital stays

A total of 757 positive samples recovered from 521 patients were included of which 400 (52.8%) progressed to death. Male patients prevailed (66.3%) but without statistical differences for gender, ICU stay, or the outcome. Male patients were also more likely to die inside ICU (34.2%). More patients received only one hospitalization (65.1%) and were also more susceptible to receive hospital discharge (33.9%; Table 1). Some patients required more than one hospital admission. Table 1 shows the basic characteristics of the studied population.

**Table 1:** Characteristics of patients admitted to the intensive care unit of a university public hospital from 2018 to 2020.

		Total *n* = 757 (%)	Hospital discharge *n* = 357 (%)	Death *n* = 400 (%)	OR (CI 95%)	*p*
Age, years (x¯ ± SD)		59.02 ± 16.13	53.99 ± 15.93	63.51 ± 14.95	-	***<0.0001*** ^*^
Gender	Male	502 (66.3)	243 (32.1)	259 (34.2)	0.86 (0.64–1.16)	*0.335^†^*
	Female	255 (33.7)	114 (15.1)	141 (18.6)		
ICU stay, days (x¯ ± SD)		22.22 ± 27.99	23.54 ± 36.74	21.05 ± 16.79	-	*0.068* ^*^
Previous hospitalization	Yes	264 (34.9)	121 (16.0)	143 (18.9)	1.08 (0.80–1.46)	*0.593^†^*
	No	493 (65.1)	236 (31.2)	257 (33.9)		

^*^Mann–Whitney test; ^†^Pearson’s chi-square (*X*^2^) test. *p* < 0.05 were considered statistically significant. x¯, mean; SD, standard deviation; CI 95%, Confidence interval 95%; OR, Odds ratio. Values in bold were considered statistically significant.

### 3.3 Scores’ results during hospital admission

The four criteria for severity of illness were not applied to all patients. The APACHE II index was obtained from 153 patients (average score of 29.1 SD ± 8.36). The highest frequency score was 32, implying that patients had a 73% chance of dying after surgical or non-surgical procedures (10). Only 95 patients were categorized according to the SOFA index (mean score of 9.59 SD ± 4.71). Most patients scored 10, representing a 40–50% chance of mortality (9). The TISS-28 index was applied to 109 patients (average score of 35.87 SD ± 8.40). The most frequent score was 42, indicating severity and the need for surveillance due to hemodynamically unstable conditions (11, 12). The BRADEN Scale was applied to 219 patients (median of 10.65). One-quarter of the patients scored >9 points; 50% scored between 9 and 12; and 25% scored <12 points. The results demonstrated a high risk of dying for most patients (12).

### 3.4 Invasive devices, comorbidity, and mortality rates

Table 2 presents the number of patients positive for MDR bacteria and the *p* values for using invasive devices (orotracheal tube, central venous catheter, double-lumen catheter, indwelling urinary catheter, and mechanical ventilation) considering the outcome and current and previous hospitalization. The use of orotracheal tube was associated with almost the double of deaths during current hospitalization (29.6%) when compared to previous hospital stay (16.0%). We observe almost the same result for those patients receiving indwelling urinary catheter (18.8% died during previous and 34.6% during current hospitalization). It is important to highlight that the previous use of mechanical ventilation was unobtainable (Table 2). This could be due to the occurrence of previous hospitalization in other medical institutions and the difficulty to consult data from other hospitals.

**Table 2:** Clinical interventions in patients during current and previous hospitalization in the intensive care unit of a university public hospital from 2018 to 2020, considering the outcome.

Previous hospital stay, *n* (%)						Current hospital stay, *n* (%)				
	Total	Death	Discharge	OR (CI 95%)	*p* ^a^	Total	Death	Discharge	OR (CI 95%)	p^*^
	*n* = 757	*n* = 400	*n* = 357			*n* = 757	*n* = 400	*n* = 357		
Orotracheal tube										
Yes	182 (24.1)	121 (16.0)	61 (8.0)	2.10 (1.48–2.97)	***<0.0001***	348 (45.8)	224 (29.6)	124 (16.4)	2.39 (1.77–3.22)	***<0.0001***
No	575 (75.9)	279 (36.9)	296 (39.1)			409 (54.2)	176 (23.2)	233 (30.8)		
Central venous catheter										
Yes	273 (36.1)	163 (21.5)	110 (14.5)	1.54 (1.41–2.08)	***0.004***	429 (56.7)	270 (35.7)	159 (21.0)	2.57 (1.93–3.49)	***<0.0001***
No	484 (63.9)	237 (31.3)	247 (32.7)			328 (43.3)	130 (17.2)	198 (26.1)		
Double-lumen catheter										
Yes	67 (8.9)	39 (5.1)	28 (3.7)	1.27 (0.77–2.08)	*0.356*	108 (14.3)	71 (9.4)	37 (4.9)	1.87 (1.21–2.86)	***0.004***
No	690 (91.1)	361 (47.7)	329 (43.5)			649 (85.7)	329 (43.4)	320 (42.3)		
Indwelling urinary catheter										
Yes	237 (31.3)	142 (18.8)	95 (12.5)	1.52 (1.11–2.07)	***0.008***	433 (57.2)	262 (34.6)	171 (22.6)	2.06 (1.53–2.77)	***<0.0001***
No	520 (68.7)	258 (34.1)	262 (34.6)			324 (42.8)	138 (18.2)	186 (24.6)		
Mechanical ventilation										
Yes	NR	NR	NR	-	-	465 (61.4)	304 (40.2)	161 (21.3)	3.85 (2.84–5.24)	***<0.0001***
No	NR	NR	NR			292 (38.6)	96 (12.7)	196 (25.9)		

^*^Pearson’s chi-square test (*X*^2^). *p* < 0.05 were considered statistically significant. OR, Odds ratio; CI 95%, Confidence interval 95%; and NR, Non recorded. Values in bold were considered statistically significant.

Table 3 compares the comorbidities detected during hospitalization and the respective outcomes. High blood pressure was the comorbidity more frequently related to in-hospital death (30.5%) followed by behavioral disorders (24.1%) and Diabetes Mellitus (20.3%). Other diseases were related to 17.2% of the outcome “death” and included neurological conditions, genetic diseases, and psychiatric illnesses.

**Table 3:** Comorbidities of patients with positive surveillance swabs in the intensive care unit of a university public hospital from 2018 to 2020, considering the outcome.

Comorbidities		Total	Death	Discharge	OR (CI 95%)	*p* ^*^
		*n* = 757 (%)	*n* = 400 (%)	*n* = 357 (%)		
High blood pressure	Yes	402 (53.1)	231 (30.5)	171 (22.6)	1.49 (1.11–1.99)	***0.007***
	No	355 (46.9)	169 (22.3)	186 (24.6)		
Behavioral disorders	Yes	320 (42.3)	182 (24.1)	138 (18.2)	1.32 (0.99–1.76)	*0.057*
	No	437 (57.7)	218 (28.8)	219 (28.9)		
Diabetes mellitus	Yes	268 (35.4)	154 (20.3)	114 (15.1)	1.33 (0.98–1.79)	0.059
	No	489 (64.6)	246 (32.5)	243 (32.1)		
Cardiac diseases	Yes	151 (20.0)	97 (12.8)	54 (7.2)	1.80 (1.25–2.60)	***0.002***
	No	606 (80.0)	303 (40.0)	303 (40.0)		
Vascular diseases	Yes	101 (13.4)	61 (8.0)	40 (5.3)	1.43 (0.94–2.17)	*0.102*
	No	656 (86.6)	339 (44.8)	317 (41.9)		
Pulmonary diseases	Yes	82 (10.8)	55 (7.2)	27 (3.6)	1.95 (1.19–3.19)	***0.006***
	No	675 (89.2)	345 (45.6)	330 (43.6)		
Chronic kidney disease	Yes	46 (6.1)	32 (4.2)	14 (1.9)	2.13 (1.12–3.97)	***0.019***
	No	711 (93.9)	368 (48.6)	343 (45.3)		
Acquired immunodeficiency syndrome	Yes	19 (2.5)	7 (0.9)	12 (1.6)	0.51 (0.20–1.24)	*0.157*
	No	738 (97.5)	393 (51.9)	345 (45.6)		
Cancer	Yes	15 (2.0)	7 (0.9)	8 (1.1)	0.78 (0.29–2.15)	*0.777*
	No	742 (98.0)	393 (51.9)	349 (46.1)		
Other diseases	Yes	240 (31.7)	130 (17.2)	110 (14.5)	1.08 (0.80–1.47)	*0.618*
	No	517 (68.3)	270 (35.7)	247 (32.6)		

^*^Pearson’s chi-square test (*X*^2^) and Fisher’s exact test. *p* < 0.05 were considered statistically significant. OR, Odds ratio; CI 95%, Confidence interval 95%. Values in bold were considered statistically significant.

### 3.5 Microbiological profile, antimicrobial use, and MDR occurrence considering the outcome

Analyzing the use of antimicrobials and the outcome, a statistical difference was observed for polypeptides (*p* < 0.004) and macrolides prescriptions and death (*p* < 0.011; Table 4). Despite being non-statically relevant, the use of β-lactams was related to 50.2% of in-hospital deaths. The drug that exhibited the second higher mortality rate was glycopeptide (36.9%) and the lowest value was observed in patients who received sulfonamides (0.9%). In-hospital deaths for patients using polypeptides corresponded to 29.5% (*n* = 223). Polypeptides also increased the risk of dying inside the ICU (OR = 1.51; CI 95% 1.14–2.03; *p* = 0.004; Table 4). Interestingly, our study demonstrated that the use of macrolides protected patients from perish inside ICU (OR = 0.57; CI 95% 0.37–0.88; *p* = 0.011).

**Table 4:** Association between antimicrobial treatment and the outcome of patients admitted to the intensive care unit of a university public hospital from 2018 to 2020.

Antimicrobials		Death	Discharge	OR (CI 95%)	*p* ^*^
		*n* = 400 (%)	*n* = 357 (%)		
β-lactam	Yes	380 (50.2)	333 (44.0)	1.36 (0.76–2.54)	*0.312*
	No	20 (2.6)	24 (3.2)		
Glycopeptides	Yes	279 (36.9)	232 (30.6)	1.24 (0.92–1.68)	*0.162*
	No	121 (16.0)	125 (16.5)		
Polypeptides	Yes	223 (29.5)	162 (21.4)	1.51 (1.14–2.03)	***0.004***
	No	177 (23.4)	195 (25.8)		
Nitroimidazoles	Yes	148 (19.6)	137 (18.1)	0.94 (0.70–1.27)	*0.697*
	No	252 (33.3)	220 (29.1)		
Aminoglycosides	Yes	77 (10.2)	68 (9.0)	1.01 (0.71–1.46)	*0.944*
	No	323 (42.7)	289 (38.2)		
Macrolides	Yes	40 (5.3)	58 (7.7)	0.57 (0.37–0.88)	***0.011***
	No	360 (47.6)	299 (39.5)		
Quinolones	Yes	37 (4.9)	24 (3.2)	1.41 (0.82–2.37)	*0.202*
	No	363(48.0)	333 (44.0)		
Lincosamides	Yes	12 (1.6)	5 (0.7)	2.17 (0.79–5.63)	*0.138*
	No	388 (51.3)	352 (46.5)		
Sulfonamides	Yes	7 (0.9)	9 (1.2)	0.69 (0.26–1.73)	*0.462*
	No	393 (51.9)	348 (46.0)		

^*^Pearson’s chi-square test (*X*^2^) and Fisher’s exact test. *p* < 0.05 were considered statistically significant. OR, Odds ratio; CI 95%, Confidence interval 95%. Values in bold were considered statistically significant.

### 3.6 Antimicrobial resistance profile of isolated MDR bacteria

**Figure 1:**
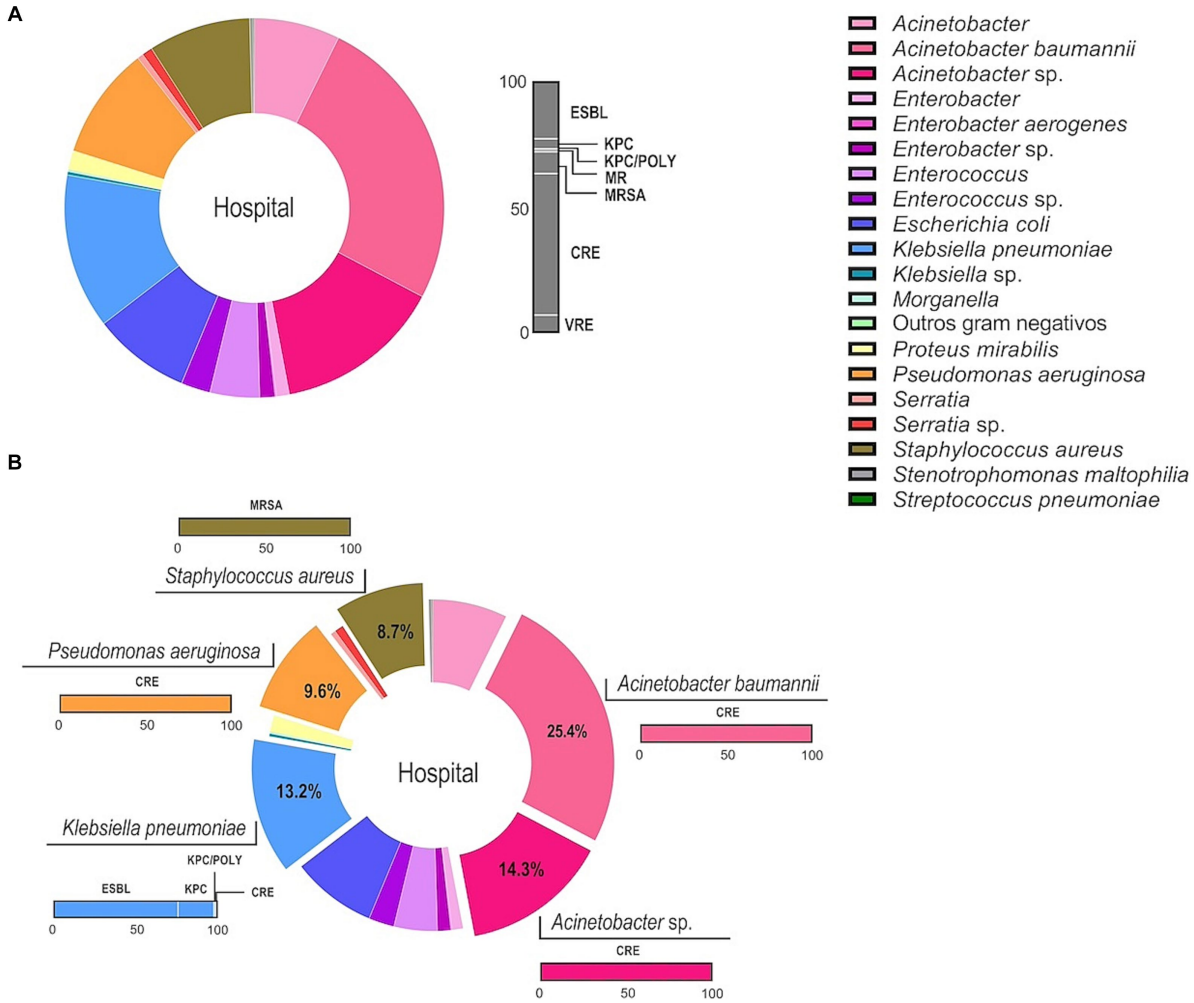
MDR profile of the microbial community recovered from ASCs in patients admitted to the intensive care unit of a university public hospital from 2018 to 2020. ESBL, Extended-spectrum beta-lactamase producing *Enterobacteriaceae*; KPC, *Klebsiella pneumoniae* carbapenemases producing; KPC/POLY, *Klebsiella pneumoniae* carbapenemases producing/polymyxin resistant strains; MR, Detection of multidrug resistant microorganism; MRSA, *Staphylococcus aureus* methicillin-resistant; CRE, Carbapenem resistant *Enterobacteriaceae*; and VRE, Vancomycin-resistant Enterococci. Data are showed as percentage and each color represent a microorganism as indicated in the legend. **(A)** Informs the total distribution of bacteria recovered from patients (left side) and their respective resistance profile to antimicrobials (right side). The gray bar represents the percentage (0–100) of all the resistance profiles observed. **(B)** The five main species and genus are highlighted, and the colored bars correspond the respective percentage of their resistance profile.

The distribution of all active surveillance cultures results and their respective antimicrobial resistance are demonstrated on Figure 1. The most frequently recovered species was *Acinetobacter baumannii* (25.4%), followed by the genus *Acinetobacter* spp. (14.3%), and other species [*Klebsiella pneumoniae* (13.2%), *Pseudomonas aeruginosa* (9.6%) and *Acinetobacter* spp., and *Pseudomonas aeruginosa* exhibited 100% CRE, *Staphylococcus aureus* was 100% MRSA, and *Klebsiella pneumoniae* isolates were 76% ESBL, 22% KPC, 1% KPC/POLY, and 1% CRE].

## 4 Discussion

### 4.1 Evaluation of ASC performance

Lower PPVs (18%) for detecting MDR bacteria in ICU patients were also observed by Ledoux et al. (14). The cost–benefit of ASC should be evaluated by the institutional biosafety committee due to its importance in investigating the microbiological profile and establishing routines to reduce and control HAIs and the emergence of MDR microorganisms. Choosing a best surveillance method adapted to the institutional characteristics is a challenge. The efficacy of the adopted methods depends on the construction, over time, of a well-structured guideline and to monitor the epidemiological profile of the hospital. As our institution is a public, university hospital and as there are national and international recommendations (15, 16) supporting the adoption of ASCs, this is the strategy we use to monitor multi-drug resistant microorganisms. Our results showed that ASC was not able to predict HAIs and open a broad discussion about its cost–benefit and to other recommendations. According to Collins (17) strategies to prevent HAIs include hand hygiene, environmental cleanliness, proper use of personnel protective equipments, antimicrobial resistance campaigns, respiratory hygiene, and cough etiquette. All these policies should be considered by our and other hospitals with the same profile. Studies discussing the impact of ASC are controversial. Some demonstrated that it decreases or eradicates MDR microorganisms combined with contact precautions (18). Other studies suggested that cohort isolation and standard precautions are more significant in preventing MDR microorganism transmission than ASCs and contact precautions for colonized patients. Despite differences, ASCs should be used when other control measures fail (15, 16).

Due to the low sensitivity of ASCs, a statistical analysis was performed considering the type of antimicrobial resistance detected. The resistance to meropenem and vancomycin was observed to be statistically significant (*p* < 0.0001). Hence, the use or recommendation of these antimicrobials indicates that patients already have or will develop HAIs. Our results corroborate with previously published data, which demonstrate that these antimicrobials are commonly used to treat HAIs, mainly caused by *A. baumannii* and *S. aureus* (19).

### 4.2 Characteristics of hospitalized patients and hospital stays

Of all patients, 53.4% were aged ≥60 years (*n* = 403), and the average age of patients who evolved to death was higher than those who received hospital discharge (*p* < 0.0001; Table 1). All hospital wards had a total of 178,635 days of hospitalization (mean of 234.1), and the ICU had 16,575 days (mean of 22.2). The length of hospital stay in all wards ranged from 1 to 1,100 days, and 50% remained hospitalized from 31 to 366.75 days. In the ICU, patients remained from 1 to 225 days (mean of 22.22 SD ± 27.99). One-quarter of the patients stayed for at least 5 days, 50% 6–23 days, and 25% up to 23 days. Patients with in-hospital death remained for fewer days in the ICU (mean of 21.05; SD ± 16.79) than those who received hospital discharge (mean of 23.54; SD ± 36.74). Our findings contradict those reported by Moraes et al. (19) who observed an association between prolonged intensive care unit (ICU) stay and the development of multidrug-resistant (MDR) microorganisms. In contrast, our data suggest that ICU stay duration may not be a significant risk factor for MDR acquisition.

### 4.3 Invasive devices, comorbidity, and mortality rates

Patients positive for MDR bacteria were frequently associated with the use of mechanical ventilation (61.4%), followed by an IUC (57.2%) and a CVC (56.7%). Mechanical ventilation was also associated with more in-hospital deaths (40.1%), followed by the use of a CVC (35.7%), an IUC (34.6%), and an orotracheal tube (29.6%). The use of an orotracheal tube had statistical significance for the outcome of “death” during previous and current hospitalization (*p* < 0.0001). During current hospitalization, all devices but the double-lumen catheter were statistically significant between survival and death groups (*p* < 0.0001; Table 2). According to Kadri et al. (20), patients admitted to the ICU have higher mortality rates and are more likely to develop HAIs than other hospital wards. In addition, these patients possess comorbidities and severe illnesses and use antimicrobials that can lead to microbial resistance (21). In-hospital deaths for patients using invasive devices ranged from 60.5% (IUC) to 65.7% (double-lumen catheter), with an average of 63.6%. The ratio between hospital discharge and in-hospital death was similar for all invasive devices (mean of 1.8), and the use of a double-lumen catheter showed slightly higher values (1.9). Using invasive devices and medical procedures in critical patients enhances the risk of infection. Higher risk is associated with deteriorating patient scenarios and a higher number of hours of healthcare assistance (22).

We observed a higher frequency of comorbidities among ICU patients, with high blood pressure prevailing, and more than one comorbidity could be diagnosed in the same patient (Table 3).

Analyzing patients’ health during ICU admission and their respective outcomes, we observed statistically associated with in-hospital death (*p* < 0.05). The risk of in-hospital death increases in the presence of HAI, invasive procedures or medical devices, the presence and severity of underlying diseases, and the adequacy of antimicrobial therapy and microorganism resistance (22). High in-hospital death rates among HAI patients are associated with comorbidities, chronic diseases, immunosuppression, and cancer (15).

### 4.4 Microbiological profile, antimicrobial use, and MDR occurrence considering the outcome

Active surveillance cultures showed a rate of 9.5% in ICU patients colonized or infected by MDR bacteria. This rate was lower than similar studies that found values between 13 and 54% (23). Mortality rates varied according to the recovered microorganism. The most lethal was *Stenotrophomona*s spp. (two patients, 100% mortality). Mortality rates ranged from 50 to 60% in the presence of *Acinetobacter* spp., *Enterococcus* spp., *Pseudomonas aeruginosa*, *Serratia* spp., and *Klebsiella pneumoniae*. The first three microorganisms presented a ratio of hospital discharge/death of 1.3–1.4. *Staphylococcus aureus* exhibited a mortality rate of 48.5%. *Escherichia coli, Enterobacter* spp., and *Proteus mirabilis* exhibited mortality rates of 42.9, 42.1, and 41.7%, respectively. Other authors mention higher mortality rates associated with MDR and Gram-negative bacteria (24, 25). It is crucial to reiterate that macrolide treatment exhibited a protective effect against in-hospital mortality, as evidenced by an odds ratio (OR) less than 1. This finding implies that individuals receiving macrolide therapy were less likely to experience in-hospital death compared to those not receiving this treatment.

### 4.5 Antimicrobial resistance profile of isolated MDR bacteria

β-lactam class was the most used group of antimicrobials (94%), followed by glycopeptides (61.4%) (8). Carbapenem resistant *Enterobacteriaceae* (CRE) was the most frequent resistance profile (*n* = 425, 56.14%), followed by extended-spectrum beta-lactamase (ESBL; *n* = 175, 23%). According to the Centers for Diseases Control and Prevention (26), CRE are considered bacteria from the order Enterobacterales resistant to at least one of the carbapenem antibiotics or produce an enzyme (carbapenemase) that can make them resistant to these medications. Carbapenemase-producing Enterobacteriaceae are a risk as carbapenems are considered a last-line of treatment against those microorganisms (27) and *Klebsiella pneumoniae* carbapenemase-producing (KPC) represented 4.7% of isolates. Gram-positive microorganisms exhibited methicillin (oxacillin)-resistant *Staphylococcus aureus* MRSA/ORSA profiles in 9.3% and vancomycin-resistant *Enterococci* (VRE) in 6.6% (Figure 1). In 2018, the CRE profile prevailed (*n* = 123), and more than half of the patients died (55%). The following year, the KPC profile prevailed, and 80% of the patients perished. In 2020, the ESBL group prevailed, and 45% of the patients died. Considering the study period, KPC and CRE profiles were related to most in-hospital deaths (55 and 45%, respectively). A statistically significant difference was not observed among all resistance profiles considering the outcome. The resistance profile within non-fermenting bacteria was in accordance with the literature (28). Our data is of great concern because the β-lactam group is considered a last-line treatment for infections caused by these pathogens, resulting in therapeutic failure and resistance to other β-lactams. This study is not without its limitations. The first is the potential interference of the COVID-19 pandemic during the sample collection period. Despite the efforts of the research team to adhere to established hospital protocols during the pandemic, it is possible that the pandemic may have influenced the study findings. The second limitation also relates to the pandemic, as it may have affected the study design. We believe that a longer data collection period would have provided a more comprehensive understanding of the results. Finally, it is important to note that our institution is a public, university-affiliated, non-profit, and philanthropic organization. As a result, our findings may reflect a local perspective. Therefore, we encourage further research in different institutions and countries to provide a broader understanding of the study topic.

## 5 Conclusion

Multidrug-resistant microorganisms represented 9.5% of all microorganisms. Gram-negative prevailed, and *A. baumannii*, *P. aeruginosa*, *K. pneumoniae*, and CRE were most frequently recovered. Among Gram-positive, vancomycin-resistant *Enterococcus* and methicillin-resistant *S. aureus* prevailed.

The use of invasive devices and antimicrobials, the length of hospital stay, the presence of MDR bacteria, and their resistance profile resulted in higher in-hospital death rates in the ICU. β-lactams and glycopeptides were the most prescribed antimicrobials and resistance to meropenem and carbapenems prevailed. The prescription of meropenem and vancomycin should be carefully monitored once patients using these antimicrobials have or will develop HAIs.

In this hospital, ASCs presented a low sensitivity and a small PPV for HAIs. Therefore, due to its importance, the institutional biosafety committee should carefully evaluate the cost–benefit of routinely using ASCs.

## Data availability statement

The original contributions presented in the study are included in the article/supplementary material, further inquiries can be directed to the corresponding author.

## Ethics statement

The studies involving humans were approved by the Educational and Extension Center of the Risoleta Tolentino Neves Hospital (NEPE/HRTN #22/2018) and the Ethics and Research Committee of the Federal University of Minas Gerais (CEP/UFMG—CAAE: 39871820.1.0000.5149). The studies were conducted in accordance with the local legislation and institutional requirements. Written informed consent for participation was not required from the participants or the participants’ legal guardians/next of kin because the information was recovered on medical records.

## Author contributions

AM: Conceptualization, Data curation, Investigation, Methodology, Writing – original draft. CM: Conceptualization, Project administration, Resources, Supervision, Writing – original draft. US: Data curation, Formal analysis, Validation, Writing – review & editing. CA: Methodology, Writing – review & editing. EL: Methodology, Writing – review & editing. LC: Validation, Writing – review & editing. PV: Writing – review & editing. CV: Data curation, Validation, Writing – original draft, Writing – review & editing. SS-K: Conceptualization, Project administration, Resources, Supervision, Funding acquisition, Writing – review & editing.
